# Synergistic Orchestration of Chlorogenic Acid Analogs in Sugarcane Molasses Polyphenols for Sucrase Inhibition: Insights into a Multilevel Inhibitory Mechanism

**DOI:** 10.3390/foods15183267

**Published:** 2026-09-16

**Authors:** Shenghong Yao, Wanlu Liu, Yumei Wang, Jiulong An, Chengfeng Zhang, Matthew Flavel, Yanv Zhou, Lu Li, He Li

**Affiliations:** 1 Key Laboratory of Geriatric Nutrition and Health, Beijing Technology and Business University, Ministry of Education, Beijing 100048, China; 2 The Product Makers Co., Ltd., Shanghai 200444, China; 3 School of Life Sciences, La Trobe University, Melbourne 3083, Australia; 4 The Product Makers (Australia), Keysborough 3173, Australia

**Keywords:** sugarcane molasses, mixed-type inhibition, synergistic effect, molecular docking, protein thermal stability, molecular dynamics simulation

## Abstract

This study systematically explored the inhibitory mechanisms against sucrase exerted by sugarcane molasses polyphenol extract (SMPE) and its three principal chlorogenic acid analogs: neochlorogenic acid (NA), chlorogenic acid (CA), and methyl chlorogenate (MC). By integrating in vitro assays, molecular simulations, and spectroscopic analyses, we demonstrated synergistic inhibition among the components. SMPE exhibited the greatest inhibitory potency (IC_50_ = 3.58 ± 0.16 mg/mL), with an IC_50_ significantly lower than those of the individual compounds. Synergy analysis indicated marked synergistic effects for both the CA-NA pair and the ternary mixture. Simultaneous multi-ligand docking further suggested complementary co-binding configurations and interactions with residues surrounding the catalytic region, providing a possible structural interpretation of the experimentally observed synergistic effects. Mechanistically, CA and NA exhibited competitive inhibition, whereas MC and SMPE showed mixed-type inhibition; fluorescence, CD, and thermal stability analyses revealed compound-dependent changes in aromatic residue microenvironments, secondary structure, and protein stability, while molecular modeling predicted peripheral binding of CA/NA and catalytic-region interactions of MC, suggesting distinct but complementary routes for perturbing substrate recognition and catalysis. This study provides mechanistic insight into the synergistic inhibition of sucrase by SMPE and supports its potential development as a natural agent for modulating postprandial glycemia.

## 1. Introduction

Inhibiting intestinal glycosidases has been a well-established approach for controlling postprandial glucose levels in diabetes management [1]. However, drugs routinely prescribed for this purpose frequently lead to gastrointestinal discomfort [2,3]. These limitations have stimulated increasing interest in plant-derived polyphenols as naturally sourced compounds for modulating carbohydrate digestion and postprandial glycemia [4,5,6]. A growing body of evidence indicates that polyphenols from tea, mulberry leaf, and other botanical materials can effectively inhibit α-glucosidase and α-amylase, pointing to their potential as natural compounds for glycemic control [7,8,9,10,11,12].

Sugarcane is one of the most widely cultivated crops worldwide, and its by-product molasses is rich in polyphenols [13,14,15]. The distribution of phenolic compounds is also markedly affected by industrial sugar processing. Payet et al. analyzed 72 samples collected from successive processing stages in the same sugar factory and reported that total phenolic concentrations increased from approximately 249 mg/kg in clarified juice to 361 mg/kg in syrup and 761–1233 mg/kg in different molasses fractions, whereas raw sugar contained only approximately 22.4 mg/kg [16]. These observations indicate that phenolic constituents become progressively enriched in the non-crystalline fractions during sugar manufacture, particularly in molasses, while only a small proportion is retained in the crystallized sugar fraction. Recently, a study has shown that sugarcane molasses-derived polyphenol-rich extract inhibited human intestinal α-glucosidases in vitro, with a strong inhibitory effect on sucrase [17]. However, acute supplementation with the extract did not significantly alter the overall postprandial glucose or insulin responses in healthy adults following a bread-based test meal. These findings indicate that the sucrase-inhibitory potential of molasses-derived polyphenols can be demonstrated in vitro, while its translation to physiological glycemic effects may depend on multiple factors. Importantly, the molecular basis underlying sucrase inhibition by molasses-derived polyphenols, particularly the contributions and potential interactions of their major phenolic constituents, remains poorly understood. A further challenge in elucidating these mechanisms arises from the high sucrose content intrinsic to molasses, which can interfere with in vitro sucrase inhibition assays and thereby obscure the inhibitory activity attributable to the polyphenolic fraction. This has hindered efforts to develop value-added applications for this abundant agricultural by-product.

In this study, we addressed this barrier by using solid-phase extraction to substantially reduce residual sucrose. We then examined the inhibitory effects of the sugarcane molasses polyphenol extract (SMPE), along with three major chlorogenic acid analogs, on sucrase activity, while also evaluating their potential synergistic interactions and underlying mechanisms of action. These findings provide a theoretical foundation for developing natural sucrase inhibitors derived from sugarcane molasses.

## 2. Materials and Methods

### 2.1. Materials and Reagents

The sugarcane molasses used in this study was supplied by The Product Makers Pty Ltd., (Keysborough, Victoria, Australia) as a by-product of industrial sugar production. In conventional sugarcane processing, juice extracted from crushed sugarcane stems is clarified, concentrated by evaporation, and subjected to vacuum crystallization, after which sucrose crystals are separated from the residual molasses fraction by centrifugation. The evaporation was carried out under vacuum at temperatures not exceeding 70 °C, and the pH during clarification was adjusted to approximately 6.0–6.5, until the Brix reached 75%. The sugarcane molasses extract was prepared from crystallized syrup using resin adsorption extraction [18]. Sucrase (from *Saccharomyces cerevisiae*, 100 U/mg), acarbose (BR, purity ≥ 95%) and chlorogenic acid (CA) were purchased from Shanghai YuanYe Biotechnology Co., Ltd. (Shanghai, China). Neochlorogenic acid (NA) and methyl chlorogenate (MC) were obtained from Chengdu Herbpurify Co., Ltd. (Chengdu, China). SYPRO^®^ Orange and DMSO (≥99.7%) were purchased from Sigma-Aldrich Chemical Co. (St. Louis, MO, USA). ISOLUTE^®^ ENV+ solid-phase extraction cartridges were purchased from Biotage AB (Uppsala, Sweden). Sucrose and potassium sodium tartrate were obtained from Fuchen Chemical Reagents Co., Ltd. (Tianjin, China). Folin–Ciocalteu reagent and 3,5-dinitrosalicylic acid (DNS) were purchased from Beijing Biotopped Technology Co., Ltd. (Beijing, China).

### 2.2. Sample Preparation

SMPE was extracted from molasses extract following a published method with slight modifications [13]. Briefly, the sugarcane molasses extract was dissolved in Milli-Q water (14 mg/mL) and centrifuged at 4000 rpm for 10 min using a refrigerated centrifuge (Allegra X-30R, Beckman Coulter, Brea, CA, USA). The supernatant was collected for subsequent purification. ISOLUTE^®^ ENV+ cartridges (sorbent: hypercrosslinked hydroxylated polystyrene–divinylbenzene copolymer; average particle size: 90 µm; pore diameter: 800 Å) were activated with methanol and equilibrated with water. Subsequently, 6 mL of the supernatant was applied to the cartridges. After loading, the cartridges were washed with 40 mL of water to remove highly water-soluble matrix constituents, particularly residual sugars. The target polyphenols were then eluted with 6 mL of methanol. The methanolic eluate was concentrated under reduced pressure using a rotary evaporator (N-1200BV, EYELA, Tokyo Rikakikai Co., Ltd., Tokyo, Japan). The resulting concentrate was finally freeze-dried for 48 h using an Alpha 1-4 LSCbasic freeze dryer (Martin Christ Gefriertrocknungsanlagen GmbH, Osterode am Harz, Germany) to obtain the polyphenol-enriched powder.

### 2.3. Determination of Residual Sucrose Content

Residual sucrose in the freeze-dried SMPE was estimated using an acid-hydrolysis method combined with the DNS assay. Briefly, SMPE was hydrolyzed with 3 M HCl at 70 °C for 15 min and subsequently neutralized with 5 M NaOH. Reducing sugar contents before and after hydrolysis were determined using the DNS method at 540 nm. Residual sucrose was calculated from the increase in reducing sugars following hydrolysis.

### 2.4. Determination of Total Polyphenol Content

The total polyphenol content was determined according to Kim et al. with minor modifications [19]. Briefly, 0.2 mL of the sample solution was combined with 1.8 mL of distilled water. Then, 0.2 mL of Folin–Ciocalteu reagent was added. The mixture was vortexed and incubated at room temperature for 5 min. Then, 2 mL of 7% sodium carbonate solution was added, and the volume was made up to 5 mL with distilled water. The reaction proceeded at room temperature for 90 min, after which the absorbance was measured at 750 nm using an Infinite 200 PRO NanoQuant microplate reader (Tecan Austria GmbH, Grödig, Austria). Gallic acid was used as the standard, and the results are expressed as mg of gallic acid equivalents (GAE) per gram of sample.

### 2.5. LC-MS/MS Analysis

Samples were analyzed with a Thermo Vanquish UHPLC system coupled to a Q-Exactive HF mass spectrometer (Thermo Fisher Scientific, Waltham, MA, USA). Separation was carried out on a Zorbax Eclipse C18 column (2.1 × 100 mm, 1.8 μm) (Agilent Technologies, Santa Clara, CA, USA) held at 30 °C. The mobile phase was composed of (A) 0.1% formic acid in water and (B) acetonitrile. A gradient elution was applied as follows: 0–2 min, 5% B; 2–6 min, linear gradient from 5% to 30% B; 6–7 min, 30% B; 7–12 min, linear gradient from 30% to 78% B; 12–14 min, 78% B; 14–17 min, linear gradient from 78% to 95% B; 17–20 min, 95% B; 20–21 min, linear gradient from 95% to 5% B; 21–25 min, 5% B. The flow rate was 0.3 mL/min, the injection volume was 2 μL, and the autosampler temperature was maintained at 4 °C. Mass spectrometric detection was conducted with the ion transfer tube temperature at 325 °C and the electrospray voltage at 3.5 kV. The sheath gas, auxiliary gas, and sweep gas flows were 45, 15, and 1 arbitrary unit, respectively. Data acquisition involved a full scan (*m*/*z* 100–1500) at a resolution of 120,000, followed by data-dependent MS/MS (dd-MS2) on the top 5 most intense ions. MS/MS spectra were generated at a resolution of 60,000 using higher-energy collisional dissociation. Data processing was performed with Compound Discoverer 3.3 for retention time correction, peak detection, and extraction. Compounds were putatively annotated based on accurate precursor masses, predicted elemental compositions, observed MS/MS fragmentation patterns, and database-assisted annotation. Spectral matching against the mzCloud and/or mzVault libraries was used when available, while ChemSpider database searching and predicted elemental compositions provided additional annotation evidence. Relative signal abundance was estimated by chromatographic peak area normalization, with the peak area assigned to each putatively annotated compound expressed as a percentage of the summed peak areas of all putatively annotated compounds.

### 2.6. Inhibition Assay of Sucrase

Briefly, 25 μL of sucrose solution (20 mg/mL) was mixed with 50 μL of samples (SMPE, CA, NA, MC or acarbose at various concentrations). The reaction was then initiated by adding 25 μL of sucrase solution (120 U/mL, prepared with 0.01 mol/L PBS, pH 6.8). The final concentrations of sucrose and sucrase in the reaction mixture were 5 mg/mL (approximately 14.6 mM) and 30 U/mL, respectively. The mixtures were incubated at 37 °C for 25 min. Then, 50 μL of DNS reagent was added, and the samples were heated in a boiling water bath for 5 min to develop the color. After cooling to room temperature, absorbance was measured at 540 nm. The inhibition rate was calculated using Equation (1). IC_50_ values were estimated by Probit regression using SPSS Statistics (version 25.0). The inhibitor concentration was log_10_-transformed for model fitting.

(1) $$Inhibition\;rate\left(\%\right)=\left[1-\frac{A_{1}-A_{2}}{A_{3}-A_{4}}\right]\times 100$$

Here, *A*_1_, *A*_2_, *A*_3_, and *A*_4_ represent the absorbance of the sample group (enzyme + polyphenol + substrate), the sample blank group (polyphenol + substrate), the control group (enzyme + substrate), and the control blank group (substrate only), respectively.

### 2.7. Molecular Docking

The crystal structure of sucrase (PDB ID: 4EQV) was downloaded from the Protein Data Bank (https://www.rcsb.org/structure/4EQV, accessed on 26 April 2025). Water molecules were removed, and hydrogen atoms were added using AutoDockTools (version 1.5.6). The 3D structures of the ligands (CA, CID: 1794427; NA, CID: 5280633; MC, CID: 6476139) were downloaded from PubChem. Ligand geometries were energy-minimized using Open Babel (version 3.1.1) with the MMFF94s force field. Hydrogen atoms were subsequently added using AutoDockTools (version 1.5.6), and the ligands were converted to PDBQT format. Molecular docking was performed using AutoDock Vina (version 1.2.7). The docking grid box was centered at x = −85.948 Å, y = 95.495 Å, and z = −78.715 Å, with dimensions of 18.75 × 37.50 × 24.00 Å along the x-, y-, and z-axes, respectively. The exhaustiveness was set to 8, and up to 9 binding poses were retained for each docking calculation. Each docking system was independently evaluated in three docking runs using different random seeds. The representative structure used for subsequent interaction analysis was selected as the lowest docking score within a consistently reproduced binding mode. For simultaneous multi-ligand docking, the corresponding ligand structures were introduced simultaneously into a single AutoDock Vina (version 1.2.7) calculation, rather than sequentially. Binary systems (CA-NA, CA-MC, and NA-MC) and the ternary system (CA-NA-MC) were evaluated using the same receptor structure, grid definition, scoring function, and docking parameters as those used for the single-ligand calculations. Visualization of docking poses was conducted using PyMOL (version 2.5.0).

### 2.8. Interaction Analysis

Interactions among polyphenolic monomers were assessed as described previously [20]. The combination ratios were based on the IC_50_ values of CA, NA, and MC. The relative sucrase activity was defined as Va, Vb, or Vc for individual polyphenols a, b, or c, respectively. For combinations, Vab and Vabc denote the observed activity with two or all three polyphenols, respectively. Assuming independent inhibitory effects, the theoretical enzyme activity (V*) for binary and ternary mixtures is Va × Vb and Va × Vb × Vc, respectively. The difference between the theoretical and observed activity was calculated as ΔV = V* − Vab (or V* − Vabc). This approach is based on the principle that combinations producing effects greater than those expected from independent action exhibit positive interactions. The classification thresholds were adopted from previous studies evaluating synergistic effects among bioactive compounds, in which deviations within ±0.10 were considered within the range of approximately additive (AD) effects, whereas deviations exceeding 0.10 were interpreted as synergistic (SY) or sub-additive (SU) interactions. Therefore, the ±0.10 criterion was used as an empirical threshold to distinguish meaningful deviations from independent responses rather than as a universal statistical boundary. Accordingly, the terms “synergistic” and “SY” used throughout this study refer to synergistic effects identified according to this predefined ΔV criterion.

### 2.9. Inhibition Kinetic Analysis

Lineweaver–Burk plots (Equation (2)) were employed to determine the inhibition modes and kinetic parameters of the polyphenols [21]. Briefly, sucrose solutions at different concentrations were mixed with varying concentrations of SMPE, CA, NA, or MC, and then sucrase (120 U/mL) was added. The reaction was conducted at 37 °C for 10 min. The amount of reducing sugars produced was then quantified by adding DNS reagent and heating the mixture at 100 °C for 5 min. After cooling, absorbance was measured at 540 nm. For each inhibitor concentration, V_max_ and K_m_ were determined by linear regression of the Lineweaver–Burk plots using GraphPad Prism (version 9.0), based on the slope (K_m_/V_max_) and y-intercept (1/V_max_) of the fitted lines. The inhibition constant (K_i_) and α for the individual polyphenols were obtained by nonlinear regression using the corresponding inhibition models in the model-fitting module of GraphPad Prism. Equations (3) and (4) correspond to the competitive inhibition model, whereas Equations (5)–(7) correspond to the mixed inhibition model.

(2) $$\frac{1}{v}=\frac{K_{m}}{V_{max}}\frac{1}{\left[S\right]}+\frac{1}{V_{max}}$$

(3) $$K_{m,obs}=K_{m}\left(1+\frac{\left[I\right]}{K_{i}}\right)$$

(4) $$v=\frac{V_{max}[S]}{K_{m,obs}+[S]}$$

(5) $$V_{max,app}=\frac{V_{max}}{1+\frac{\left[I\right]}{\alpha K_{i}}}$$

(6) $$K_{m,app}=K_{m}\frac{1+\frac{\left[I\right]}{K_{i}}}{1+\frac{\left[I\right]}{\alpha K_{i}}}$$

(7) $$v=\frac{V_{max,app}[S]}{K_{m,app}+[S]}$$

The following kinetic parameters are used: the reaction rate (v), the maximum reaction rate (V_max_), the Michaelis constant (K_m_), the substrate concentration ([S]), and the inhibitor concentration ([I]). For inhibition studies, apparent parameters are defined. In competitive inhibition, the apparent Michaelis constant is denoted as K_m,obs_. In mixed/non-competitive inhibition, the apparent maximum rate and Michaelis constant are V_max,app_ and K_m,app_, respectively. α describes the inhibitor’s relative affinity for the free enzyme versus the enzyme–substrate complex. α = 1 indicates equal affinity for both forms; α > 1 signifies preferential binding to the free enzyme; and α < 1 indicates preferential binding to the enzyme–substrate complex.

### 2.10. Fluorescence Spectroscopy Analysis

#### 2.10.1. Intrinsic Fluorescence Emission

Fluorescence measurements were performed as described previously using an FS5 fluorescence spectrophotometer (Edinburgh Instruments Ltd.,Livingston, Scotland, UK) [22]. Sucrase was mixed with SMPE, CA, NA, or MC in a fixed volume ratio (1:1), with the inhibitor concentration varying across a specified range. The fluorescence intensity was recorded at 37 °C. With an excitation wavelength of 280 nm, the emission spectra were collected from 300 to 400 nm. The fluorescence quenching data were analyzed using the Stern–Volmer equation (Equation (8)). A linear Stern–Volmer plot indicates the presence of a single quenching mechanism, either static or dynamic. When both static and dynamic quenching occur simultaneously, the plot exhibits an upward curvature. In such cases, the data were analyzed using the sphere-of-action model [23], which assumes the existence of a spherical region around the fluorophore where quenching occurs with uniform probability. This model is described by the modified Stern–Volmer equation (Equation (9)).

(8) $$\frac{F_{0}}{F}=1+k_{q}\tau_{0}\left[Q\right]=1+K_{sv}[Q]$$

(9) $$\frac{F_{0}}{F}=\left(1+K_{app}\left[Q\right]\right)exp(\left[Q\right]VN/1000)$$

Here, F_0_ and F represent the maximum fluorescence intensities of the enzyme in the absence and presence of polyphenols, respectively; k_q_ is the bimolecular quenching constant; τ_0_ is the fluorescence lifetime in the absence of a quencher; K_sv_ is the Stern–Volmer quenching constant; and [Q] is the polyphenol concentration. K_app_ denotes the apparent static quenching constant, V is the volume of the quenching sphere, and N is Avogadro’s number.

#### 2.10.2. Synchronous Fluorescence Measurements

Synchronous fluorescence spectroscopy was used to probe the microenvironment of tyrosine and tryptophan residues in sucrase. Spectra were acquired at constant wavelength differences (Δλ) of 15 nm (for tyrosine) and 60 nm (for tryptophan). The ratio of synchronous fluorescence quenching (RSFQ) was calculated using Equation (10).

(10) $$RSFQ=\left(1-\frac{F}{F_{0}}\right)$$

Here, F_0_ and F are the synchronous fluorescence intensities of sucrase in the absence and presence of polyphenols, respectively.

### 2.11. Circular Dichroism (CD) Spectroscopy

Circular dichroism spectra were recorded on a CD spectrometer (MOS-500, Bio-Logic SAS, Seyssinet-Pariset, France) as described previously with minor modifications [22]. Briefly, sucrase was incubated with each polyphenol (SMPE, CA, NA, or MC) at varying concentrations while maintaining a 1:1 volume ratio. Spectra were acquired from 190 to 260 nm with a 0.5 nm step size and 2 s per point. CDNN (version 2.1) was used to analyze the secondary-structure content of sucrase.

### 2.12. Thermal Stability Analysis

The thermal stability of sucrase in the presence of polyphenols was evaluated by differential scanning fluorometry (DSF) using an UNcle protein stability analysis system (Uncle, Unchained Labs, Pleasanton, CA, USA). Following a reported procedure with minor modifications [24], sucrase was mixed with each polyphenol and then with an equal volume of SYPRO^®^ Orange dye (20×). A 9 μL aliquot of the mixture was loaded into Unis (Unchained Labs) for analysis. The temperature was ramped up from 25 °C to 90 °C at a linear rate of 0.5 °C/min. Throughout the increase, full-spectrum fluorescence (250–720 nm) was continuously recorded under 473 nm laser excitation [25]. The melting temperature and related parameters were derived using the UNcle Analysis software (Version 6.01).

### 2.13. Molecular Dynamics Simulation

Molecular dynamics simulations of sucrase and the sucrase–polyphenol complexes were performed using GROMACS 2023. The initial sucrase structure was obtained from the Protein Data Bank (PDB ID: 4EQV), and the structures of CA, NA, and MC were obtained from PubChem (CID: 1794427, 5280633, and 6476139, respectively). Ligand topology files were generated using Sobtop (version 1.0), and MMFF94 atomic partial charges were assigned to the ligands. The protein was described using the Amber14SB force field. The sucrase and sucrase–polyphenol complexes were placed in dodecahedral simulation boxes solvated with TIP3P water molecules, and sodium and chloride ions were added to neutralize the total system charge. Energy minimization was performed using 10,000 steps of steepest descent followed by 5000 steps of conjugate-gradient minimization. After energy minimization, the systems were equilibrated for 200 ps under the NVT ensemble and subsequently for 200 ps under the NPT ensemble. The temperature and pressure were maintained at 310 K and 1 bar using the V-rescale thermostat and Parrinello–Rahman barostat, respectively. Production molecular dynamics simulations were then carried out for 100 ns, with coordinates saved every 10 ps. Each system was subjected to a single 100 ns production trajectory. Stabilization of the RMSD profile was used to assess whether the trajectory had reached a relatively stable conformational regime before subsequent structural interpretation. RMSD, RMSF, radius of gyration (Rg), and solvent-accessible surface area (SASA) were calculated from the production trajectories using the corresponding GROMACS analysis utilities. Free energy landscapes were constructed with RMSD and Rg as collective variables using a custom Python (version 3.7) script.

### 2.14. Data Analysis

Unless otherwise stated, all biochemical and spectroscopic experiments, including the Folin–Ciocalteu assay, enzyme inhibition and kinetic assays, fluorescence spectroscopy, circular dichroism spectroscopy, and differential scanning fluorometry, were performed in three independent experimental replicates. Data are presented as mean ± standard deviation (SD). Analysis of variance (ANOVA) and Duncan’s multiple range test (*p* < 0.05) were utilized to determine the statistical significance of differences. Graphical plots were generated using GraphPad Prism version 9.0 or Origin 2025.

## 3. Results and Discussion

### 3.1. Polyphenolic Profile of SMPE

Under the laboratory-scale SPE conditions used in this study, approximately 3.8 g of freeze-dried SMPE was obtained from 14.0 g of molasses extract, corresponding to an approximate dry-extract yield of 27.1% (*w*/*w*). The preparation consumed approximately 1.0 L of methanol and 6.67 L of water for this amount of starting material. The total phenolic content of the freeze-dried extract, determined using the Folin–Ciocalteu assay, was 259.3 ± 5.7 mg GAE/g dry extract. UHPLC-MS/MS analysis detected numerous compounds in SMPE, from which 27 polyphenolic compounds were selected for further annotation and characterization. Detailed information on the precursor ions, major MS/MS fragment ions, annotation sources, and available mzCloud and mzVault Best Match values is provided in Table S1. Among these 27 selected polyphenolic compounds, the compounds putatively annotated as NA, CA, and MC exhibited the highest relative LC-MS peak areas, accounting for 36.36%, 25.70%, and 18.04%, respectively, and collectively representing approximately 80.10% of the summed peak area of the 27 selected polyphenolic compounds. Because MS response factors can vary among chemically distinct compounds owing to differences in ionization efficiency and matrix effects, these normalized peak area percentages should not be interpreted as absolute mass or molar composition [26]. Additionally, the residual sucrose content of the SMPE, estimated by the acid-hydrolysis/DNS method, was approximately 0.62 ± 0.01% (*w*/*w*), confirming that the SPE purification effectively removes the vast majority of sugars and that the observed sucrase inhibition is attributable to the polyphenolic constituents rather than to any residual substrate interference. Based on their high relative LC-MS peak areas, NA, CA, and MC were selected as representative major candidate compounds for subsequent functional evaluation to investigate their potential contributions to the sucrase-inhibitory activity of SMPE.

### 3.2. Elucidation of the Inhibitory Mechanism of Polyphenols Against Sucrase Through Molecular Docking

#### 3.2.1. Inhibitory Effects of Polyphenols on Sucrase

The inhibitory activities of SMPE and its major polyphenolic constituents (CA, NA, MC) against sucrase were assessed. The results demonstrated that all compounds inhibited sucrase activity (Figure S1). SMPE was the most potent, with an IC_50_ of 3.58 ± 0.16 mg/mL, significantly lower than those of the individual compounds. Among the three pure compounds, NA was the most potent inhibitor (IC_50_ = 5.90 ± 0.17 mg/mL), while CA was the weakest (IC_50_ = 8.01 ± 0.14 mg/mL). As a reference inhibitor, acarbose exhibited an IC_50_ of 36.18 ± 1.35 mg/mL. Under the experimental conditions used in this study, the tested polyphenols exhibited substantial inhibitory activity against sucrase. Notably, the inhibitory potency of SMPE as a polyphenol mixture surpasses that of any single component. This phenomenon aligns with reports on polyphenols from honeysuckle berries and edible seaweed [27,28]. Thus, the superior activity of SMPE likely stems from synergistic interactions among its polyphenolic components.

**Figure 1:**
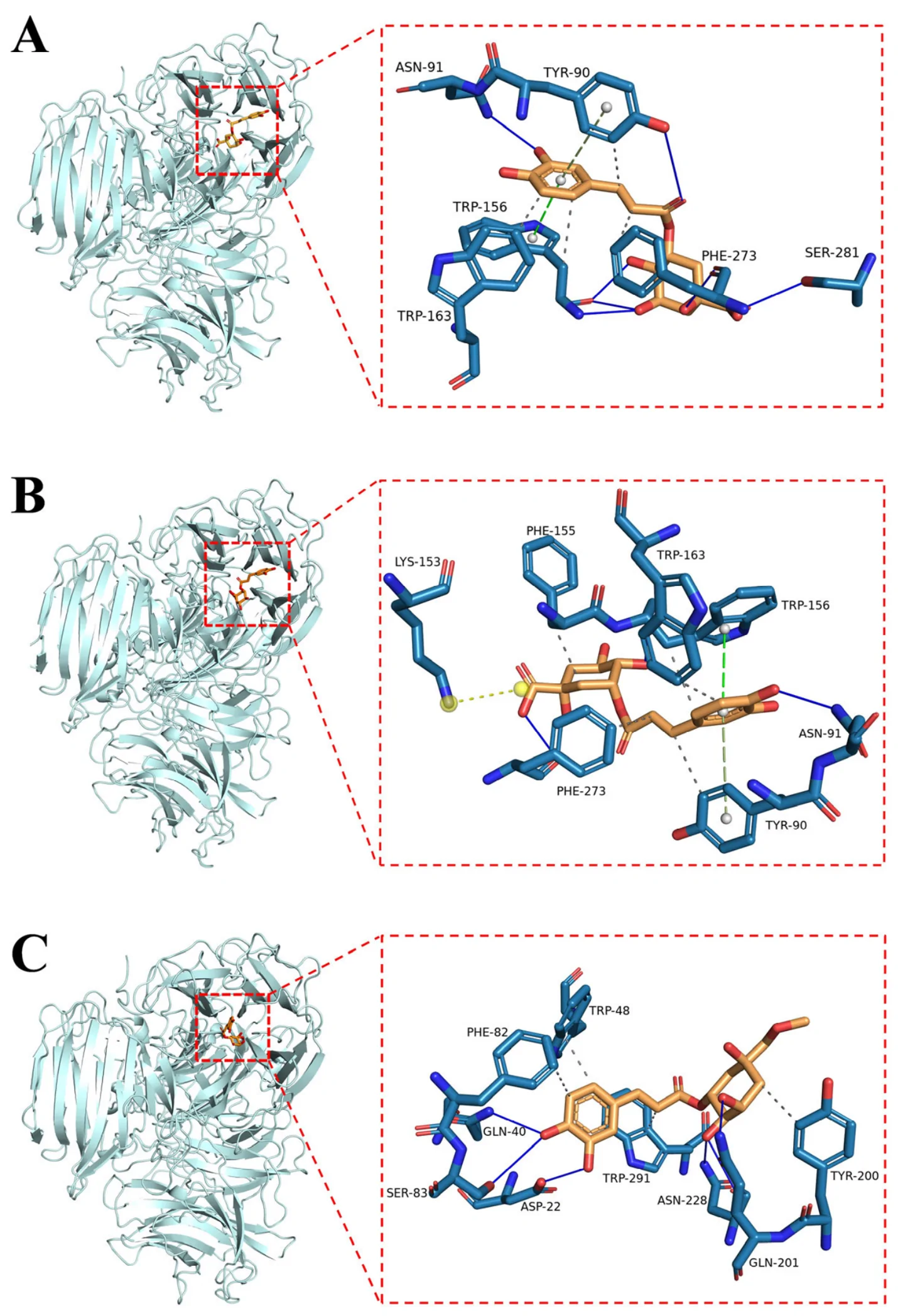
Interaction modes of sucrase with chlorogenic acid (**A**), neochlorogenic acid (**B**), and methyl chlorogenate (**C**) at their minimum Vina docking scores. The interactions include hydrophobic interactions (gray dashed lines), hydrogen bonds (blue solid lines), π-π stacking (dark green dashed lines), and salt bridges (yellow dashed lines).

To elucidate the structural basis for the observed differences in inhibitory activity, molecular docking was used to simulate the binding modes of the three chlorogenic acid analogs (CA, NA, and MC) with sucrase. The docking results for CA, NA, and MC are presented in Figure 1A–C. The inhibitory effects of polyphenols are known to be strongly influenced by their structural features, particularly the number and spatial arrangement of phenolic hydroxyl and carboxyl groups [29]. All three compounds are esterification products of caffeic acid and quinic acid, but differ in their substitution patterns: CA is esterified at the 3-hydroxyl of the quinic acid moiety, NA at the 5-hydroxyl, and MC is the methyl ester derivative of CA. As shown in Figure 1 and Table S2, the caffeoyl moieties of both CA and NA adopt a similar binding orientation, forming interactions with residues Trp90, Asn91, and Trp156. However, the differing esterification positions alter the spatial orientation of the quinic acid moiety, thereby modulating its specific interactions with surrounding residues in the active site. Although hydrogen bonding is known to be crucial for polyphenol-mediated enzyme inhibition [30], our docking results (Table S2) reveal a paradox: NA, which forms fewer hydrogen bonds with sucrase (three) than CA (seven), is the more potent inhibitor. The number of hydrophobic interactions is comparable (five for NA vs. four for CA). The docking model provides a structural explanation for this discrepancy. The unique orientation of NA’s quinic acid moiety places its carboxyl group near the ε-amino group of the lysine residue (Lys153), facilitating the formation of a salt bridge that may favor ligand–enzyme interactions [31,32]. This predicted interaction may contribute to NA’s greater inhibitory potency compared with CA. These findings underscore that, in addition to hydrogen bonding, other noncovalent interactions such as salt bridges may play an important role in determining the inhibitory potency of polyphenol–enzyme complexes. In addition, although MC is structurally similar to CA, the docking results revealed notable differences in both their binding sites and interaction patterns. MC yielded a less favorable Vina docking score than CA. However, MC interacted with several key residues, including the nucleophilic catalytic residue Asp22 and hydrophobic pocket residues such as Trp48, Phe82, and Trp291 [33]. These interactions may inhibit the catalytic hydrolysis of the substrate and alter its positioning within the active pocket, thereby reducing enzyme activity both directly and indirectly. Therefore, the more potent inhibitory effect of MC compared with CA may be attributed to its closer proximity to the catalytic center, enabling more effective interactions with these key residues. Collectively, these findings suggest that polyphenols inhibit sucrase with varying strengths, reflecting their distinct binding modes and interaction mechanisms.

#### 3.2.2. Analysis of Interactions Among Polyphenols

Given their distinct binding modes, we hypothesized that combining the polyphenols might yield synergistic inhibitory effects. We therefore assessed the inhibitory activities of all pairwise and ternary combinations of CA, NA, and MC. Owing to their different interactions with sucrase, the combinations were classified as sub-additive (SU), additive (AD), or synergistic (SY) according to their deviation from the theoretical response expected under independent action [34]. The combined inhibitory effects at different concentration ratios are summarized in Table 1. Under the predefined ΔV criterion, most tested combinations were classified as synergistic, and the CA-NA pair showed the largest positive ΔV values. At a low CA concentration (4 mg/mL), increasing NA from 3 to 6 mg/mL enhanced the synergistic effect. In contrast, the CA-MC combination primarily exhibited additive or weakly synergistic effects. Despite these relatively modest interaction effects, the overall inhibitory effect generally increased with increasing concentrations of either component, with a more consistent increase observed when the CA concentration was raised. The NA-MC pair elicited SU effects at lower concentrations, but these were gradually attenuated as the concentration of either polyphenol increased, eventually shifting toward synergistic behavior. Moreover, the ternary mixture of CA, NA, and MC consistently produced SY effects, which may partly contribute to the lower IC_50_ observed for SMPE relative to the individual compounds. Notably, while the NA-MC pair alone showed only SU or AD interactions at low concentrations, the inclusion of CA converted their combined effect into a stronger SY response, underscoring the critical role of CA in enabling cooperative inhibition among the polyphenols. It should be noted that different interaction models address different aspects of combination effects. The ΔV approach applied here evaluates whether the observed inhibition exceeds the theoretical response expected from independent action, which is suitable for identifying potential cooperative effects among selected polyphenolic combinations. Chou-Talalay CI and isobolographic approaches provide complementary quantitative frameworks by integrating dose–response relationships across multiple effect levels [35,36]. Therefore, the synergistic interactions identified in this study represent enhanced inhibitory effects relative to independent expectations under the predefined ΔV criterion, while additional dose–response-based analyses could further characterize the quantitative strength of these interactions.

**Figure 2:**
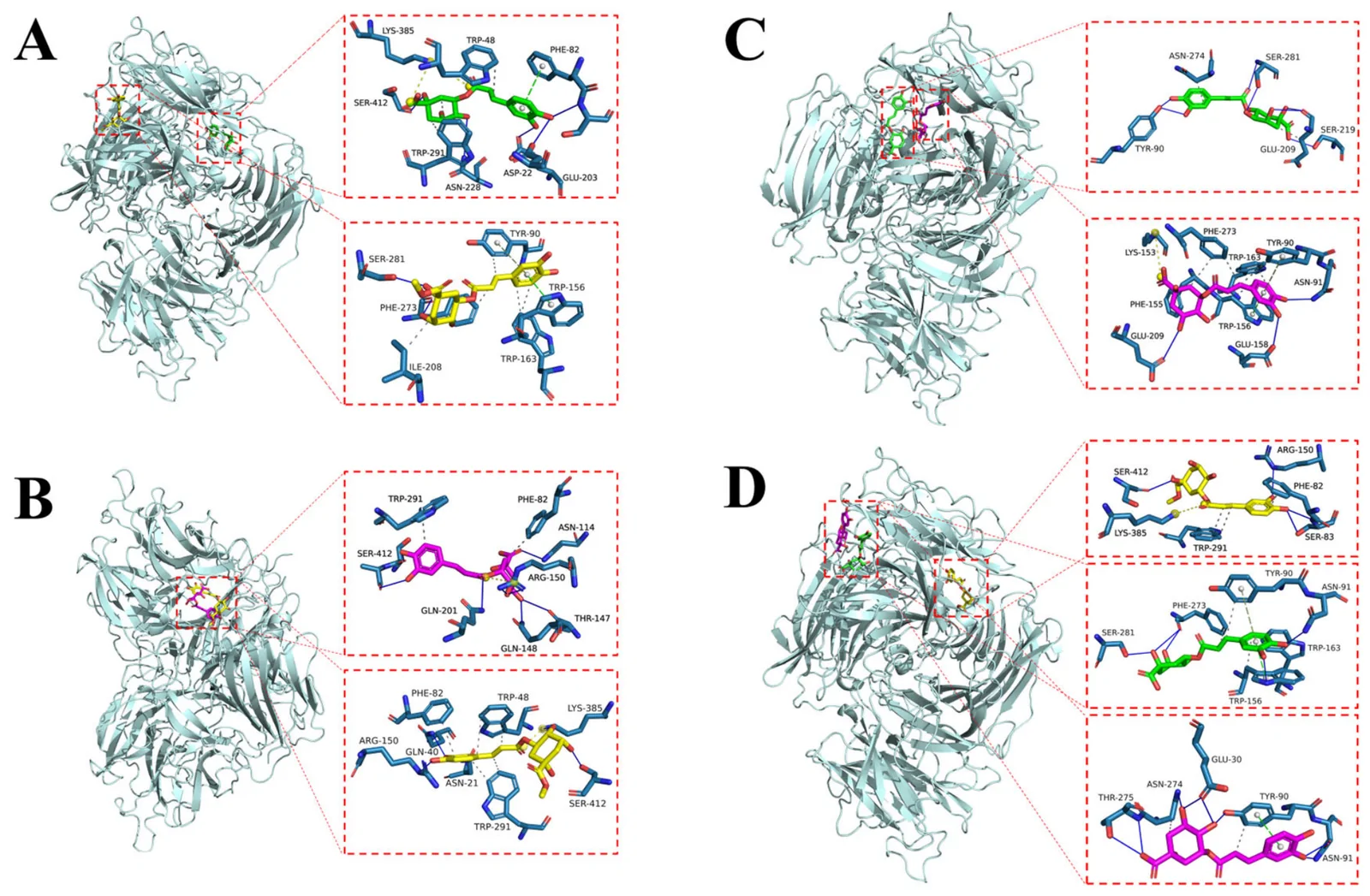
Interaction modes of sucrase with chlorogenic acid (CA) and methyl chlorogenate (MC) (**A**), CA and neochlorogenic acid (NA) (**B**), NA and MC (**C**), and CA, NA, and MC (**D**) in the conformations with the lowest Vina docking score. CA is shown in green, MC in yellow, and NA in purple. The interactions include hydrophobic interactions (gray dashed lines), hydrogen bonds (blue solid lines), π-π stacking (dark green dashed lines), and salt bridges (yellow dashed lines).

To elucidate the molecular basis of the polyphenols’ synergistic inhibition, we performed multi-ligand docking to simulate their simultaneous binding to sucrase. As shown in Figure 2A, CA and MC bind in the vicinity of the enzyme’s five-bladed β-propeller catalytic domain. Notably, the binding mode engaged additional critical residues, including the catalytic acid/base Glu203 and the peripheral active-site residue Ser412 [33], which are not efficiently targeted by the individual polyphenols. These additional interactions likely contribute to the enhanced inhibitory effect of the combination. In contrast, CA and NA were found to bind in closer proximity to each other within the catalytic region of sucrase (Figure 2B). As shown in Tables S2 and S3, NA maintained its characteristic interaction profile in the complex, while CA provided supplementary hydrogen bonds. The predicted co-binding pose therefore suggests that CA may complement the interaction network of NA by providing additional hydrogen bonds near the catalytic center, which could contribute to the synergistic effect observed experimentally. As for the combination of NA and MC, the binding sites are located closer to the catalytic pocket (Figure 2C). As indicated in Tables S2 and S3, the pair engaged hydrophobic residues (Trp48, Phe82, and Trp291) and the peripheral residue Ser412 via hydrophobic interactions and hydrogen bonds. Notably, unlike MC alone, the NA-MC pair did not contact the catalytic nucleophile. The predicted co-binding configuration suggests that simultaneous occupancy by NA and MC could interfere with substrate access and optimal positioning within the catalytic pocket, rather than directly contacting the catalytic residues. This provides a possible structural explanation for their concentration-dependent combination effects observed experimentally. Figure 2D illustrates the binding mode when all three chlorogenic acid analogs (CA, NA, and MC) were docked simultaneously. The top-ranked ternary co-binding configuration yielded a Vina docking score of −14.0 kcal/mol. In this predicted configuration, MC was positioned closest to the catalytic center, whereas CA and NA were located near the central axis of the β-propeller fold. MC formed hydrophobic interactions with Trp291, hydrogen bonds with Ser412, and a salt bridge with Lys385 (Table S3). These complementary predicted interactions suggest a plausible co-binding arrangement through which the three chlorogenic acid analogs may jointly perturb substrate positioning and the local catalytic environment. Together, the in vitro combination assays provide experimental evidence for synergistic inhibition, while the multi-ligand docking models suggest plausible complementary co-binding interactions that may help explain the observed combination effects at the structural level.

#### 3.2.3. Inhibition Kinetics of Polyphenols Against Sucrase

**Figure 3:**
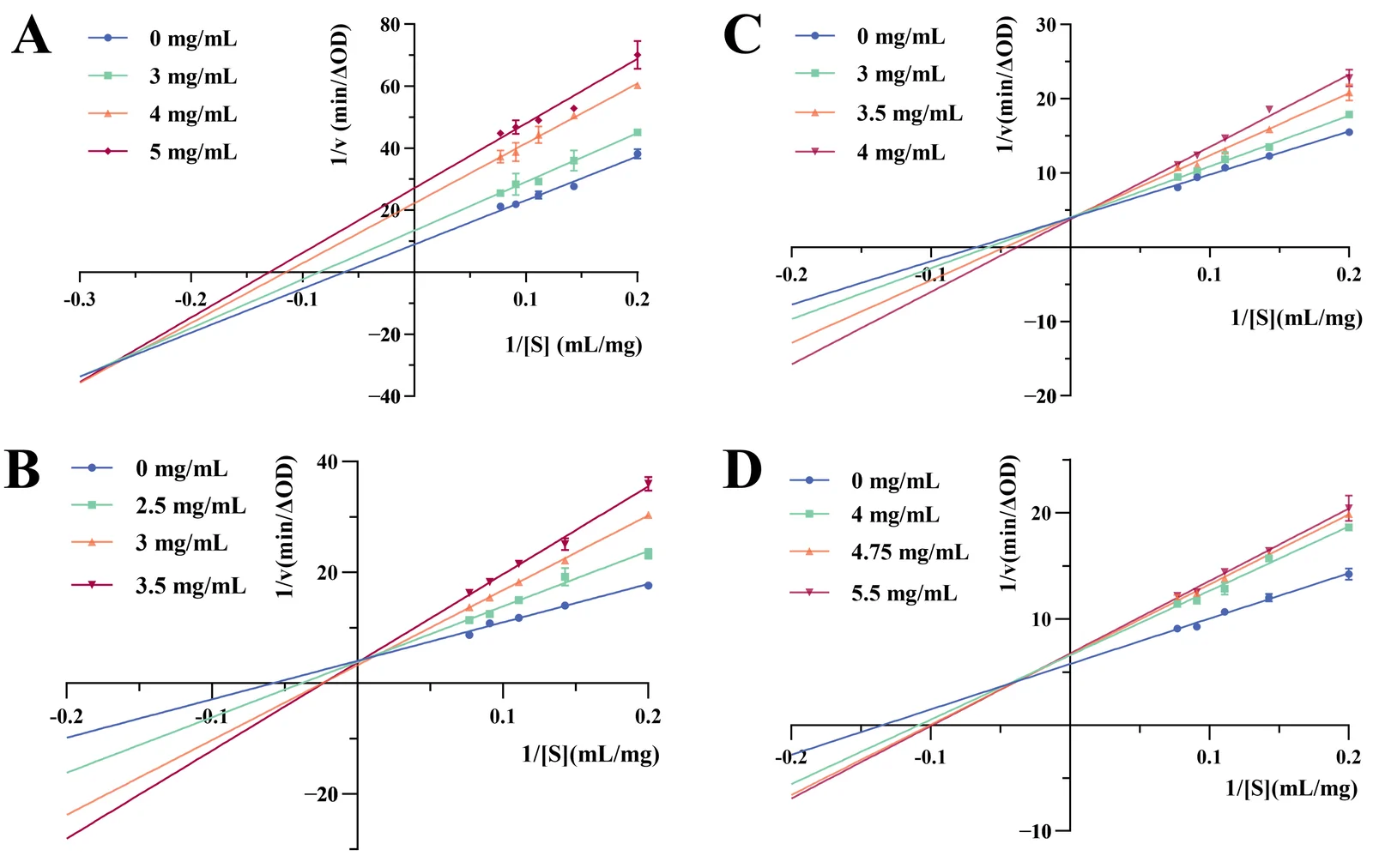
In vitro inhibition kinetics of sucrase by polyphenols. Lineweaver–Burk plots of sucrase in the presence of sugarcane molasses polyphenol extract (**A**), neochlorogenic acid (**B**), chlorogenic acid (**C**), and methyl chlorogenate (**D**).

To further elucidate the inhibitory mechanisms, enzyme kinetics were analyzed using Lineweaver–Burk plots. SMPE exhibited a mixed-type inhibition pattern, as evidenced by lines intersecting in the third quadrant and concurrent decreases in both V_max_ and K_m_ with increasing SMPE concentration (Figure 3A; Table S5). In contrast, CA and NA showed competitive inhibition, with the fitted lines intersecting on the *Y*-axis (Table S4). Increasing inhibitor concentrations resulted in an increase in the apparent K_m_, whereas V_max_ remained nearly constant (Figure 3B,C), supporting a competitive kinetic relationship with sucrose. Molecular docking predicted that CA and NA bind at regions adjacent to, rather than directly occupying, the catalytic center. One possible interpretation is an allosterically coupled competitive mechanism, in which ligand binding at a spatially distinct site induces conformational changes that reduce productive substrate binding, thereby making substrate-bound and inhibitor-bound states functionally mutually exclusive. Such a mechanism can produce a competitive kinetic pattern without direct occupation of the substrate-binding site [37]. The inhibition constant (K_i_), which reflects inhibitor–enzyme affinity [38], was lower for NA (3.489 ± 0.369 mg/mL) than for CA (9.788 ± 1.021 mg/mL), indicating stronger binding by NA. This ranking is consistent with their respective in vitro IC_50_ values, suggesting that the lower K_i_ of NA may contribute to its greater inhibitory potency relative to CA. For MC, the Lineweaver–Burk plots showed lines intersecting in the second quadrant, with V_max_ decreasing and K_m_ increasing as its concentration rose, characteristic of mixed-type inhibition (Figure 3D). An α value greater than 1, together with the increase in K_m_, suggests that MC binds more readily to the free enzyme than to the enzyme–substrate complex [39]. Although MC’s K_i_ (12.160 ± 2.008 mg/mL) was higher than that of CA, it was a more potent inhibitor in vitro. This indicates that, in addition to binding affinity, the binding mode of the inhibitor with either the free enzyme or the enzyme–substrate complex is also a crucial factor influencing its overall inhibitory potency. Furthermore, although both SMPE and MC exhibited mixed-type inhibition, their binding characteristics differed: the fitted mixed inhibition model suggested an apparent preference of SMPE-associated inhibition for the enzyme–substrate state relative to the free-enzyme state, whereas MC showed the opposite tendency. This distinction may be attributed to two factors. First, combination and docking analyses suggest that the chlorogenic acid analogs in SMPE may engage a broader set of amino acid residues, potentially producing more extensive interaction networks that could influence the enzyme differently in its free and substrate-bound states. Second, minor polyphenolic constituents in SMPE may contribute to inhibition through additional mechanisms, collectively amplifying the uncompetitive inhibition behavior observed for SMPE [40].

### 3.3. Fluorescence Spectroscopy Analysis of the Interaction Between Polyphenols and Sucrase

Having established the inhibitory modes of the polyphenols, we next investigated whether their binding induces conformational changes in sucrase. We first used fluorescence spectroscopy to probe potential alterations in the microenvironment of aromatic amino acid residues upon polyphenol binding.

#### 3.3.1. Intrinsic Fluorescence Spectroscopy

**Figure 4:**
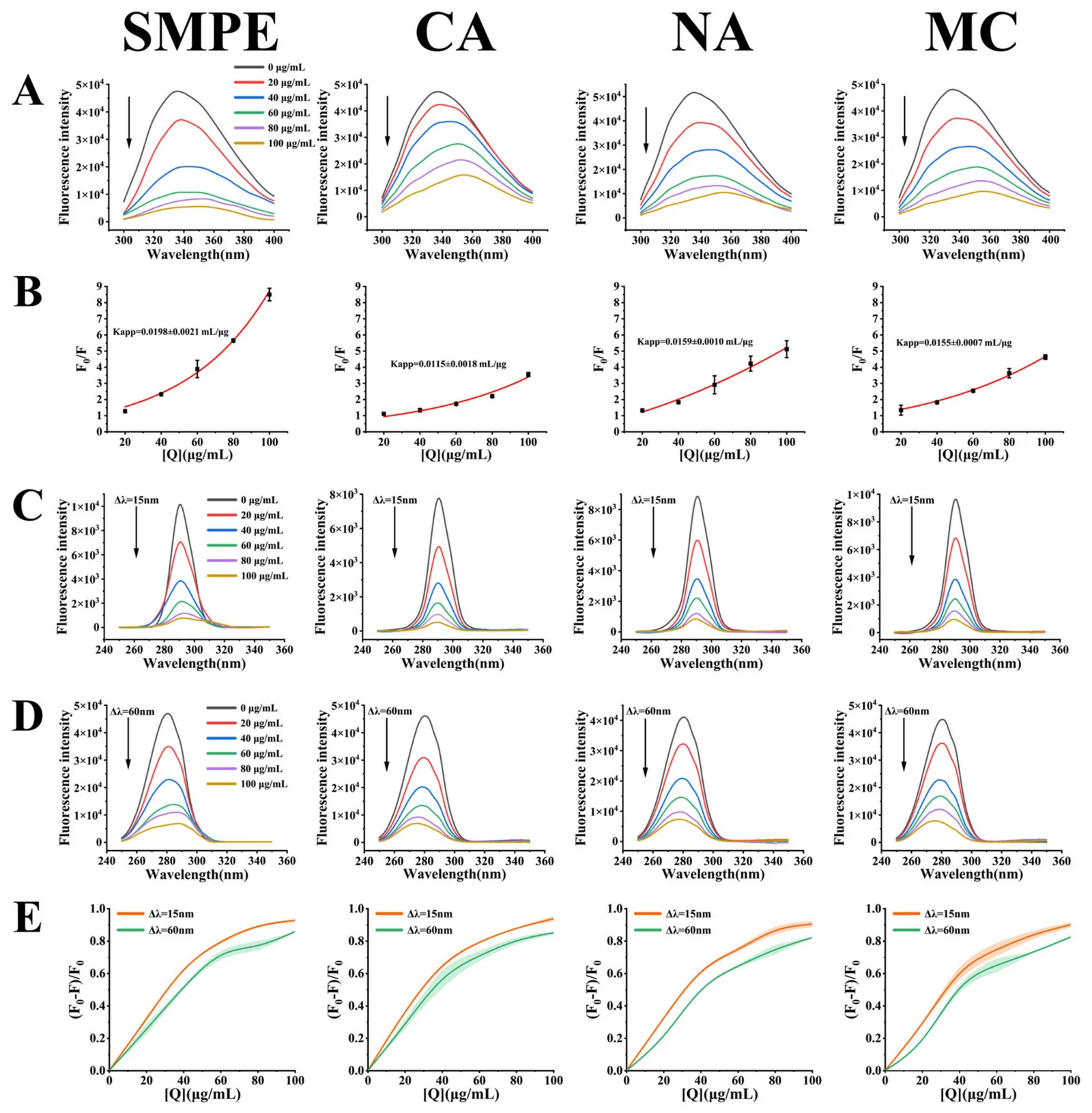
Fluorescence spectroscopic analysis of sucrase treated with sugarcane molasses polyphenol extract (SMPE), chlorogenic acid (CA), neochlorogenic acid (NA), and methyl chlorogenate (MC) (from left to right), showing: intrinsic fluorescence quenching spectra (**A**); Stern–Volmer fitting curves (**B**); synchronous fluorescence spectra at Δλ = 15 nm (**C**) and Δλ = 60 nm (**D**); and synchronous fluorescence quenching ratio plots (**E**). Black arrows indicate the direction of fluorescence intensity changes with increasing polyphenol concentration.

The intrinsic fluorescence of sucrase originates from its aromatic amino acid residues, whose emission intensity is highly sensitive to the local microenvironment of the chromophores [41]. When small molecules bind to proteins, the fluorescence quenching effect can be used to reveal the interaction mode between polyphenols and the enzyme [42]. We recorded the intrinsic fluorescence spectra of sucrase with increasing concentrations of each polyphenol. As shown in Figure 4A, fluorescence intensity decreased progressively with increasing SMPE concentrations, indicating quenching due to binding between SMPE and the enzyme fluorophores. A noticeable redshift in the emission maximum was also observed, suggesting a slight local unfolding of the enzyme and increased polarity around the fluorophores [43]. These fluorescence changes indicate that SMPE alters the local microenvironment of aromatic fluorophores, particularly by increasing their exposure to a more polar environment. When considered together with the docking-predicted interactions near the catalytic region, such local conformational perturbations may contribute to impaired substrate recognition or positioning. Each polyphenol also induced a redshift in the emission maximum (CA: 337 → 355 nm; NA: 336 → 357 nm; MC: 336 → 356 nm), indicating increased polarity around the fluorophores. This shift likely results from the exposure of aromatic residues to a more polar solvent environment through hydrophobic interactions and hydrogen bonding with the polyphenols.

Fluorescence quenching of sucrase by the four polyphenols was analyzed using the Stern–Volmer equation. The upward-curving plots of F_0_/F versus [Q] (Figure 4B) indicate that both static and dynamic quenching occur simultaneously [23,44]. This suggests that the polyphenols interact with the enzyme not only through multiple noncovalent interactions but also through free diffusion collisions. The apparent quenching constants (Kapp) decreased in the following order: SMPE (0.0198 ± 0.0021 mL/µg) > NA (0.0159 ± 0.0010 mL/µg) > MC (0.0155 ± 0.0007 mL/µg) > CA (0.0115 ± 0.0018 mL/µg). This ranking agrees with the in vitro inhibition results, corroborating that SMPE is the most efficient inhibitor [45].

#### 3.3.2. Synchronous Fluorescence Spectroscopy

To complement the intrinsic fluorescence data and probe polyphenol binding to specific aromatic residues, we employed synchronous fluorescence spectroscopy. This technique selectively targets tyrosine and tryptophan residues by using fixed wavelength intervals (Δλ) of 15 nm and 60 nm, respectively [46,47]. The interaction between SMPE and sucrase is shown in Figure 4C,D. With increasing concentrations, all compounds quenched the fluorescence of both Tyr and Trp residues. SMPE induced a clear redshift in the Trp emission peak (281 → 288 nm), indicating an increase in microenvironment polarity [47], consistent with the intrinsic fluorescence results. In contrast, the monomeric polyphenols (CA, NA, and MC) caused a blue shift in the Trp peaks (CA: 281 → 276 nm; NA: 280 → 278 nm; MC: 280 → 276 nm), while the Tyr peaks remained largely unaffected. This blue shift suggests that these monomers promote the burial of Trp residues into a more hydrophobic local environment [47], likely through hydrogen bonding, hydrophobic interactions, and π-π stacking. Together with the molecular docking results, these local environmental changes may reflect ligand-induced conformational rearrangements that could influence substrate access to the catalytic region. The ratio of synchronous fluorescence quenching data (Figure 4E) revealed that at 100 μg/mL, SMPE quenched 92.77 ± 0.89% of Tyr residue fluorescence and 85.80 ± 0.28% of Trp residue fluorescence. A similar preference for quenching Tyr over Trp residues was observed for the three individual polyphenols. This indicates that the polyphenols are spatially closer to Tyr residues and interact more readily with Tyr residues [48].

### 3.4. Analysis of Conformational Changes in Sucrase Induced by Polyphenols Using Circular Dichroism Spectroscopy

**Figure 5:**
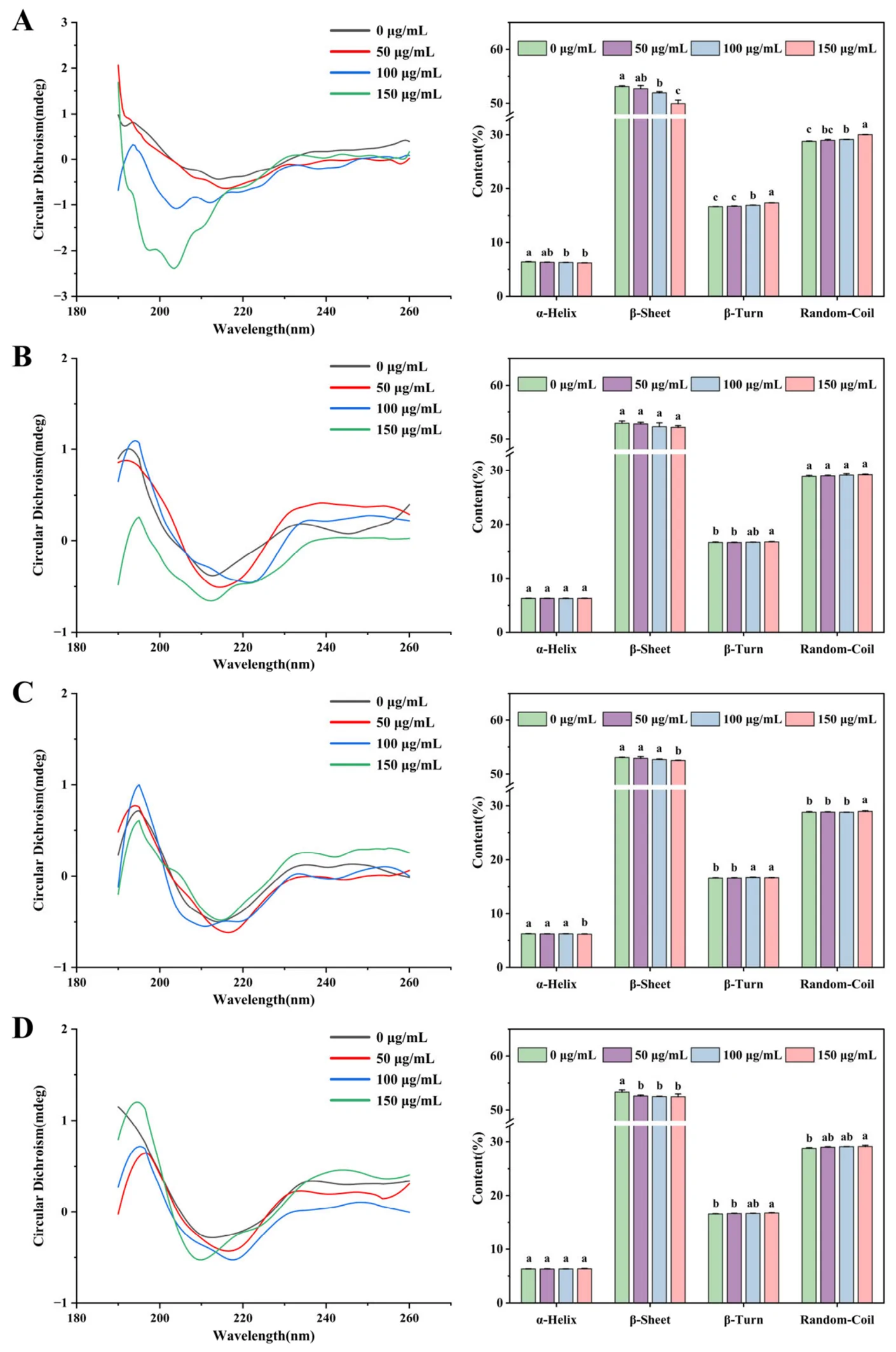
Circular dichroism spectra of sucrase after treatment with different concentrations of sugarcane molasses polyphenol extract (**A**), chlorogenic acid (**B**), neochlorogenic acid (**C**), and methyl chlorogenate (**D**) (left) and the corresponding changes in secondary-structure content (right). Different lowercase letters indicate significant differences among different concentrations within the same secondary structure (*p* < 0.05).

While fluorescence spectroscopy confirmed interactions between polyphenols and aromatic residues, the consequent effects on sucrase’s global structure required clarification. We therefore employed circular dichroism spectroscopy to estimate changes in secondary-structure composition, thereby linking structural perturbations to the observed loss of enzymatic activity. Figure 5A–D show the CD spectra of sucrase in the presence of SMPE, CA, NA, and MC. SMPE induced the most pronounced structural perturbation, characterized by a significant decrease in α-helix and β-sheet content alongside an increase in β-turn and random coil (Figure 5A). This indicates a partial unfolding or conformational loosening of the enzyme [49], consistent with the intrinsic fluorescence results. NA induced similar but less dramatic changes than SMPE (Figure 5C), correlating with its slightly lower inhibitory potency. MC also notably reduced β-sheet content (Figure 5D). In contrast, CA produced minimal alterations to the secondary structure (Figure 5B). It has been reported that the β-propeller structure consists of multiple repeated antiparallel β-sheets, and the catalytic domain of sucrase is primarily composed of β-propeller structures [50]. The decreases in β-sheet content and corresponding increases in less ordered structural components suggest changes in the overall conformational framework of the enzyme. For the individual polyphenols, docking models further predict interactions with residues located in or near the catalytic region, providing a possible structural context through which these broader conformational changes may influence substrate recognition and catalysis. In contrast, CA had a minimal effect on the secondary structure of sucrase; aside from changes in β-turn content, the proportions of other structures remained essentially unchanged. This disparity likely stems from the distinct binding modes and locations of the individual polyphenols within the enzyme [51]. Collectively, the CD results demonstrate that polyphenol binding is accompanied by changes in the global secondary structure of sucrase. In combination with the docking and kinetic results, these conformational changes may contribute to the observed inhibition by modifying the structural environment required for efficient substrate recognition and catalysis.

### 3.5. Thermal Stability Analysis of Sucrase by Differential Scanning Fluorometry

**Figure 6:**
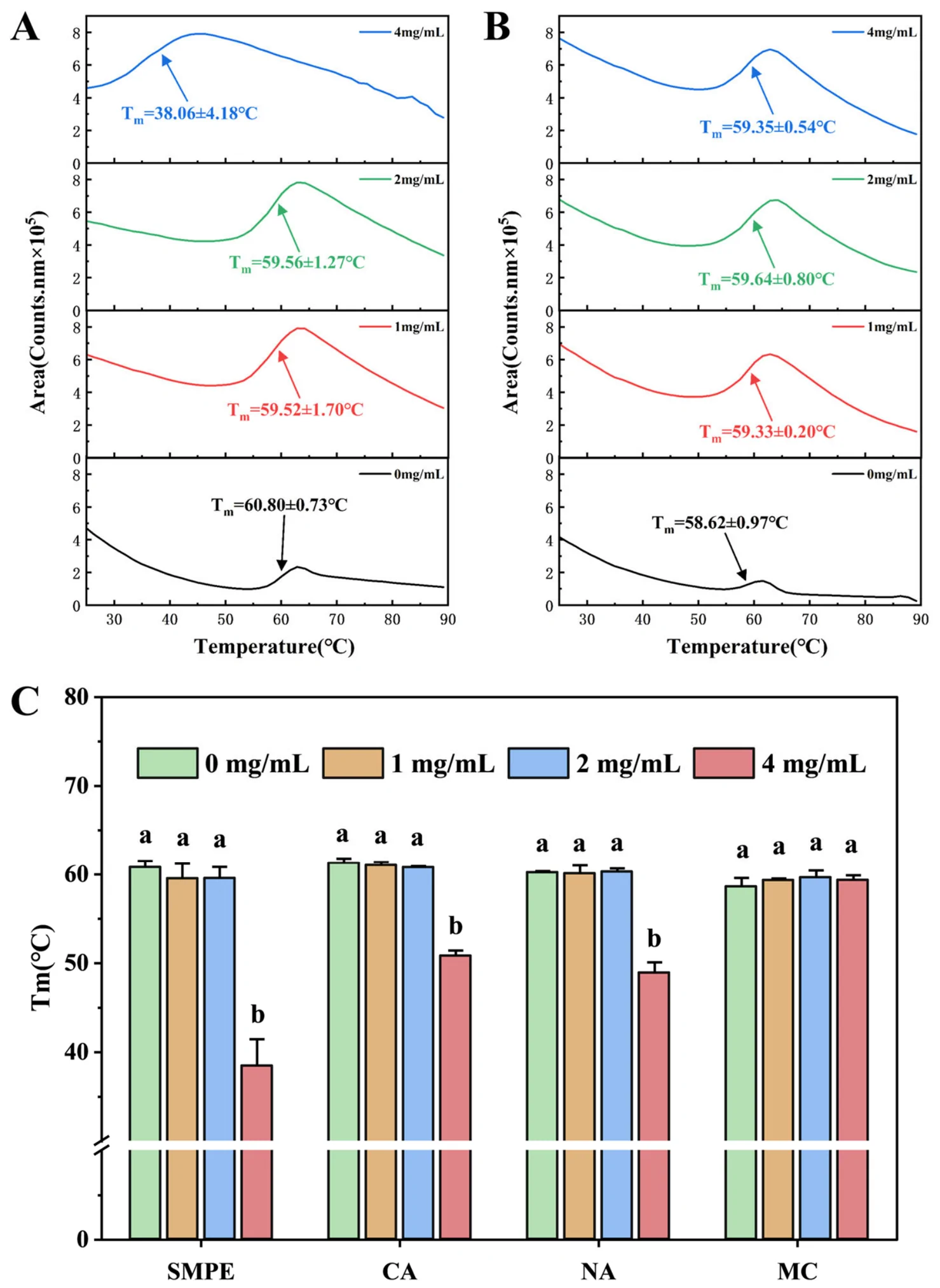
Thermal denaturation analyses of sucrase following polyphenol binding. Fluorescence peak area versus temperature curves for sucrase treated with different concentrations of sugarcane molasses polyphenol extract (SMPE) (**A**). Corresponding thermal denaturation profiles for sucrase treated with methyl chlorogenate (MC) (**B**). Melting temperature (T_m_) values of sucrase after incubation with four concentrations of SMPE, chlorogenic acid (CA), neochlorogenic acid (NA), and MC (**C**). Lowercase letters indicate statistically significant differences (*p* < 0.05) among concentrations within each polyphenol group.

Having shown that polyphenol binding alters the local microenvironment of aromatic residues and the global secondary structure of sucrase, we subsequently used differential scanning fluorometry to assess its effects on protein thermal stability. In DSF, the SYPRO^®^ Orange dye binds to hydrophobic patches exposed during thermal denaturation, leading to an increase in fluorescence [25]. The temperature at which the fluorescence peak area increases most rapidly corresponds to the protein’s melting temperature (T_m_). A decrease in T_m_ signifies reduced thermal stability, which often reflects a departure from the optimally folded, catalytically competent native state under physiological conditions, potentially leading to activity loss [52]. As shown in Figure 6A, SMPE markedly reduced the thermal stability of sucrase, lowering its melting temperature from 60.80 ± 0.73 °C to 38.06 ± 4.18 °C at 4 mg/mL. Similarly, CA and NA also significantly decreased T_m_, though to a lesser extent than SMPE at the same concentration (Figure S2). It has been proposed that allosteric inhibitors can reduce thermal stability by inducing conformational changes and domain movements [53]. Given that the catalytic pocket of sucrase is formed by β-propeller and β-sheet structures whose activity depends on their precise arrangement [33], we propose that the binding of CA or NA induces subtle shifts within the β-propeller domain. This allosteric mechanism would not only directly contribute to inhibition but also account for the observed decrease in thermal stability. Additionally, previous results indicated that SMPE, CA, and NA caused redshifts in the enzyme’s fluorescence peaks, consistent with sucrase unfolding and decreased thermal stability [54]. In contrast, Figure 6B,C show that MC at the same concentration did not substantially alter sucrase’s T_m_, indicating minimal impact on thermal stability, even though a redshift in fluorescence and significant inhibition of enzyme activity were observed. This phenomenon can be explained as follows: First, the fluorescence redshift reflects changes in the local microenvironment and solvent exposure of aromatic fluorophores. Among all amino acid residues interacting with MC, aromatic residues account for approximately 44.4%, which is lower than that of CA (66.7%) and NA (71.4%). The interaction with a more diverse set of amino acid residues may explain why MC induces only a redshift in fluorescence without causing a significant change in T_m_ [54]. Second, ligands with higher binding affinity may cause larger T_m_ shifts in DSF [55]. MC’s lower affinity (higher K_i_) and mixed-type inhibition profile align with its modest effect on thermal stability. Taken together, MC likely inhibits sucrase through direct interactions at the catalytic site without inducing extensive global conformational disruption, a mechanism which resembles that reported for tannic acid inhibition of α-amylase [56]. In summary, methylation of the carboxyl group in MC’s quinic acid moiety alters its binding pattern with sucrase relative to CA, thereby altering its binding affinity and inhibitory potency, accounting for the differential effects on the enzyme’s T_m_.

### 3.6. Molecular Dynamics Simulation of Polyphenol Binding to Sucrase

**Figure 7:**
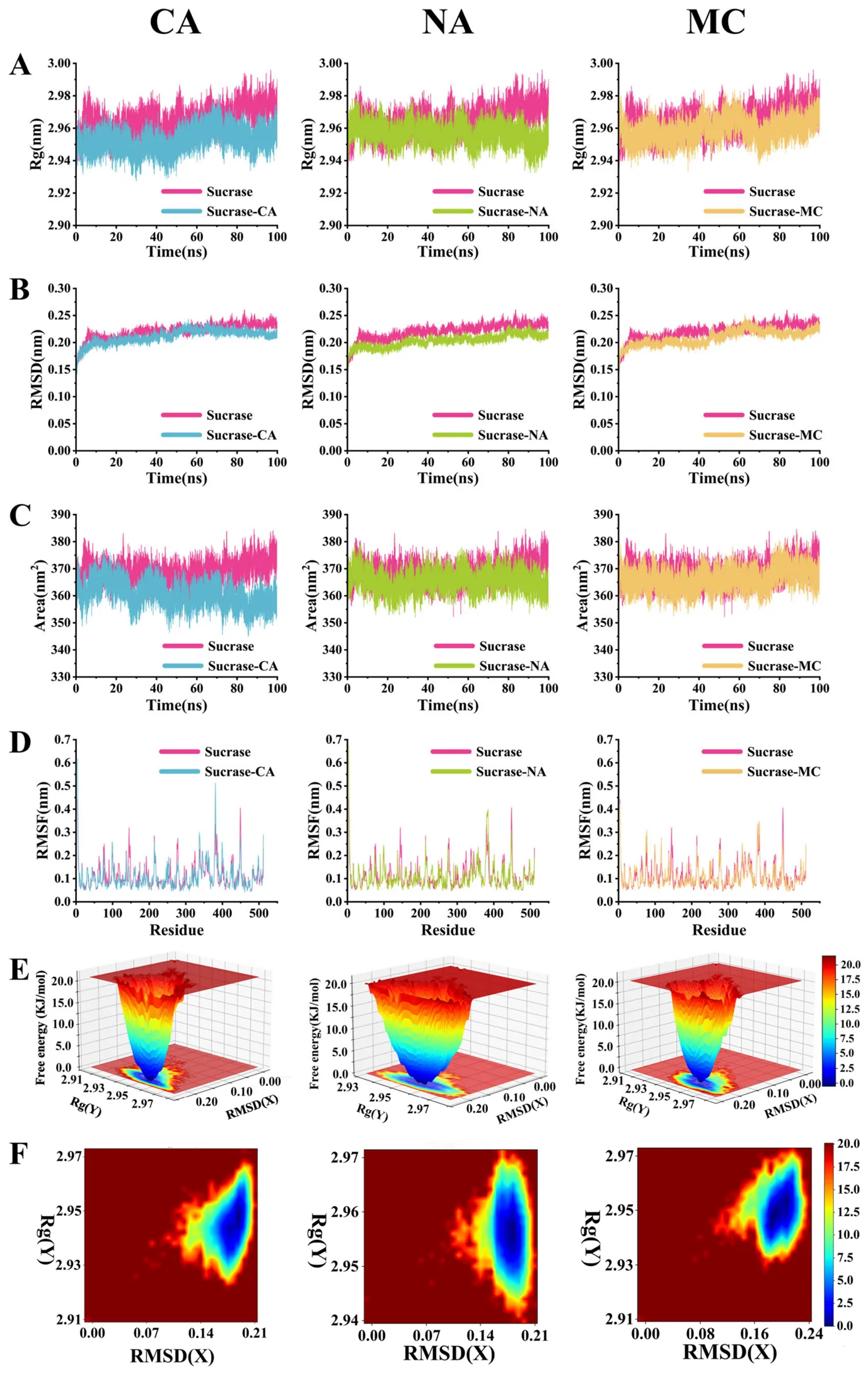
Structural dynamics of sucrase in complex with: chlorogenic acid (CA), neochlorogenic acid (NA), and methyl chlorogenate (MC). Time-dependent structural parameters: radius of gyration (**A**), root mean square deviation (**B**), and solvent accessible surface area (**C**). Root mean square fluctuation per residue (**D**). Three-dimensional (**E**) and two-dimensional (**F**) free energy landscapes.

The spectroscopic and thermal stability data demonstrated that polyphenol binding induces dynamic conformational changes in sucrase. To model these processes at atomic resolution and evaluate the stability of the predicted binding modes, we conducted 100 ns molecular dynamics simulations. The radius of gyration (Rg), a parameter defined as the root mean square distance of atoms from the protein center of mass that reflects structural compactness [57], was calculated for each system. As shown in Figure 7A, after binding with the three polyphenols, the Rg values of sucrase were generally lower than those of the enzyme alone, with the decrease being relatively smaller in the case of MC. Within the 100 ns trajectories examined, the ligand-bound systems generally exhibited lower Rg values than the enzyme, suggesting a trend toward increased structural compactness during the simulated timescale. The root mean square deviation (RMSD) was employed to evaluate conformational stability throughout the simulation trajectories [58]. The sucrase–NA complex showed the lowest RMSD values among the three ligand-bound systems during the sampled trajectory, suggesting comparatively smaller global structural deviations over the 100 ns simulation period, while the CA- and MC-bound complexes exhibited moderately higher deviations. The RMSD profiles entered relatively stable fluctuation ranges after approximately 10 ns for the CA complex and approximately 5 ns for the NA and MC complexes (Figure 7B). Notably, the MC complex showed increased fluctuation around the 40 ns mark. Although CA formed more hydrogen bonds with the enzyme, the quinic acid moiety in NA adopts a spatial orientation that enables the formation of a predicted salt bridge with Lys153, which may contribute to the comparatively lower RMSD observed for the NA complex during the simulated trajectory. This structural observation aligns well with NA’s greater inhibitory potency. As shown in Figure 7C, all three polyphenols reduced the solvent accessible surface area (SASA) of sucrase to varying degrees, with the sucrase–CA complex showing the lowest SASA value, indicating that CA induced a greater degree of folding in the enzyme. Root mean square fluctuation (RMSF) analysis was used to examine local residue flexibility [57]. Most residues in the three enzyme–polyphenol complexes exhibited RMSF values below 0.1 nm (Figure 7D), suggesting relatively limited local fluctuations during the sampled trajectories [59]. In addition, Table S6 reveals that NA engaged a higher percentage of residues in stabilizing interactions (71.43%) compared with CA (66.67%), which is consistent with the comparatively smaller local fluctuations observed in the NA-bound trajectory. Notably, MC maintained stable contacts with key catalytic residues Asp22 and Trp291, supporting its proposed mechanism of inhibition through direct interaction with residues crucial for substrate hydrolysis.

The free energy landscape (FEL), constructed using RMSD and Rg as conformational coordinates, provides an intuitive representation of the conformational space sampled during the simulations. In Figure 7E,F, the color gradient represents the energy states of each system, with deep red indicating higher relative free energy regions and deep blue corresponding to lower relative free energy regions. The three enzyme–polyphenol complexes exhibited well-defined low-energy basins in the conformational space, indicating that the trajectories were predominantly distributed within relatively restricted conformational regions. Together with the Rg and synchronous fluorescence results, these conformational features suggest that polyphenol binding may promote a more constrained conformational state of the enzyme, potentially affecting substrate access to the catalytic region [60]. Notably, the MC–sucrase complex displayed two distinct low-energy basins, suggesting that the system sampled two major conformational states during the 100 ns trajectory [61].

## 4. Conclusions

The present findings indicate that synergistic interactions among NA, CA, and MC make an important contribution to the overall sucrase-inhibitory activity of SMPE. As a mixture, SMPE exhibits significantly more potent inhibition than any of its single components. Mechanistically, SMPE functions overall as a mixed-type inhibitor. At the same time, its individual components display distinct inhibition modes: CA and NA exhibited competitive inhibition kinetics, while molecular docking suggested that their binding at non-catalytic regions may influence substrate recognition through conformational coupling. The lower fitted K_i_ of NA, indicating stronger apparent inhibitory affinity under the kinetic model, may be partly associated with its docking-predicted salt-bridge interaction with Lys153. MC displays mixed-type inhibition, likely through interactions with catalytic residues such as Asp22 and the hydrophobic pocket. The combination assays showed that the three enzyme–polyphenol combinations exhibited enhanced inhibition under the predefined ΔV criterion. Multi-ligand docking further suggested complementary co-binding configurations that may interfere with substrate positioning and catalysis. Spectroscopic and thermal stability analyses further indicate that SMPE and the representative polyphenols can alter the local environment of aromatic residues and, to varying degrees, the global conformation and thermal stability of sucrase. When considered together with the docking and molecular dynamics results, these structural perturbations may contribute to altered interactions within or around the catalytic region. In contrast, MC does not significantly alter the overall spatial structure of the enzyme, yet still effectively suppresses enzymatic function through specific local interactions.

Several limitations should be considered when extrapolating these findings to physiological or functional-food applications. First, the inhibitory activity was evaluated using *S. cerevisiae* invertase, which, although a well-established model for investigating sucrose hydrolysis and glycosidase inhibition, is not identical to mammalian intestinal sucrase–isomaltase. Therefore, the present findings provide preliminary evidence for the potential of sugarcane molasses polyphenols to interfere with sucrose digestion, but cannot be directly interpreted as definitive inhibition of human intestinal sucrase. In addition, gastrointestinal digestion and interactions with the food matrix may alter the stability and release of the polyphenols, while intestinal absorption and metabolism may further influence the chemical forms and concentrations present at the intestinal site of action. It should be noted that the concentrations used in the biochemical assays were selected to establish concentration–response relationships and elucidate inhibitory mechanisms and should not be interpreted as direct estimates of phytochemical concentrations achievable in the human intestinal lumen. Accordingly, the intestinal exposure levels of NA, CA, MC, and other SMPE constituents after oral intake remain unknown. The dose feasibility and gastrointestinal tolerability of SMPE at functionally relevant intake levels also remain to be established. From a processing perspective, the present SPE procedure was developed for laboratory-scale preparation of SMPE rather than as an optimized production process. The relatively high solvent-to-feed ratio associated with the small-scale cartridge format would require substantial optimization before practical scale-up. Larger packed-bed or reusable resin systems may offer potential routes to improve throughput, reduce solvent consumption, and enable adsorbent regeneration. Methanol was used as the elution solvent in the present laboratory procedure; however, for future food-oriented applications, more food-compatible solvent systems, such as ethanol or aqueous ethanol, should be evaluated with respect to phenolic recovery, selectivity, and biological activity. Solvent recovery and recycling should also be considered to reduce solvent consumption, environmental burden, and production cost. Future studies incorporating gastrointestinal digestion, mammalian or human intestinal sucrase–isomaltase models, intestinal cellular systems, animal experiments, and ultimately controlled human studies are required to establish physiological relevance, effective intestinal exposure, tolerability, and practical feasibility as a functional ingredient. In parallel, process optimization and scale-up studies will be necessary to evaluate food-compatible solvent systems, solvent and resin recycling, production efficiency, and the practical feasibility of SMPE as a functional ingredient.

## Figures and Tables

**Table 1:** Effects of different combinations of chlorogenic acid (CA), neochlorogenic acid (NA), and methyl chlorogenate (MC) at varying concentrations on sucrase inhibition.

Group	Va + Vb	Value			Interaction Effect
		Vab	V*^1^	V*^1^ − Vab	
1	4 mg/mL CA + 3 mg/mL NA	0.28778	0.58367	0.29589	SY ^I^
2	4 mg/mL CA + 6 mg/mL NA	0.06546	0.40056	0.33509	SY
3	8 mg/mL CA + 3 mg/mL NA	0.05640	0.35643	0.30003	SY
4	8 mg/mL CA + 6 mg/mL NA	0.01430	0.24461	0.23031	SY
5	4 mg/mL CA + 3.6 mg/mL MC	0.58964	0.62711	0.03747	AD ^II^
6	4 mg/mL CA + 7.2 mg/mL MC	0.29049	0.40939	0.11890	SY
7	8 mg/mL CA + 3.6 mg/mL MC	0.12156	0.38296	0.26140	SY
8	8 mg/mL CA + 7.2 mg/mL MC	0.02661	0.24867	0.22206	SY
9	3 mg/mL NA + 3.6 mg/mL MC	0.89714	0.54600	−0.35110	SU ^III^
10	3 mg/mL NA + 7.2 mg/mL MC	0.50433	0.35643	−0.14790	SU
11	6 mg/mL NA + 3.6 mg/mL MC	0.28598	0.37470	0.08872	AD
12	6 mg/mL NA + 7.2 mg/mL MC	0.11310	0.24461	0.13151	SY
Group	Va + Vb + Vc	Vabc	V*^2^	V*^2^ − Vabc	Interaction Effect
13	4 mg/mL CA + 3 mg/mL NA + 3.6 mg/mL MC	0.12781	0.44705	0.31923	SY
14	4 mg/mL CA + 3 mg/mL NA + 7.2 mg/mL MC	0.04297	0.29184	0.24886	SY
15	4 mg/mL CA + 6 mg/mL NA + 3.6 mg/mL MC	0.02635	0.30679	0.28044	SY
16	4 mg/mL CA + 6 mg/mL NA + 7.2 mg/mL MC	0.01533	0.20028	0.18495	SY
17	8 mg/mL CA + 3 mg/mL NA + 3.6 mg/mL MC	0.02196	0.27300	0.25103	SY
18	8 mg/mL CA + 3 mg/mL NA + 7.2 mg/mL MC	0.01418	0.17822	0.16403	SY
19	8 mg/mL CA + 6 mg/mL NA + 3.6 mg/mL MC	0.01351	0.18735	0.17384	SY
20	8 mg/mL CA + 6 mg/mL NA + 7.2 mg/mL MC	0.00889	0.12230	0.11342	SY

^I^ SY represents a synergistic effect identified according to the predefined ΔV criterion; ^II^ AD represents an additive effect; ^III^ SU represents a sub-additive effect.

## Data Availability

The original contributions presented in this study are included in the article/Supplementary Materials. Further inquiries can be directed to the corresponding author.

## Supplementary Materials

The following supporting information can be downloaded at https://www.mdpi.com/article/10.3390/foods15183267/s1: Figure S1: Comparison of IC_50_ values of sucrase for four polyphenols and acarbose. Figure S2: Fluorescence peak area of sucrase as a function of temperature after interaction with different concentrations of CA (A) and NA (B). Table S1: UHPLC-MS/MS-based putative annotation of selected polyphenolic compounds in SMPE. Table S2: Binding sites and interaction types of the three polyphenols with sucrase. Table S3: Binding sites and interaction forces when multiple polyphenols are simultaneously docked with sucrase. Table S4: Kinetic inhibition parameters of sucrase in the presence of CA, NA, and MC. Table S5: Kinetic inhibition parameters of sucrase in the presence of SMPE. Table S6: RMSF values of selected amino acid residues in sucrase before and after binding with polyphenols.

## Abbreviations

The following abbreviations are used in this manuscript:

AD	Additive
CA	Chlorogenic acid
CD	Circular dichroism
DNS	3,5-dinitrosalicylic acid
DSF	Differential scanning fluorometry
FEL	Free energy landscape
GAE	Gallic acid equivalents
MC	Methyl chlorogenate
NA	Neochlorogenic acid
Rg	Radius of gyration
RMSD	Root mean square deviation
RMSF	Root mean square fluctuation
SASA	Solvent accessible surface area
SMPE	Sugarcane molasses polyphenol extract
SU	Sub-additive
