# Supplementary material for: Synergistic Orchestration of Chlorogenic Acid Analogs in Sugarcane Molasses Polyphenols for Sucrase Inhibition: Insights into a Multilevel Inhibitory Mechanism
Source: Foods. 2026 Sep 16;15(18):3267. doi: 10.3390/foods15183267 (PMC13606313; doi:10.3390/foods15183267)
Supplement: Supplementary file 1 [file foods-15-03267-s001.zip › foods-4503980-supplementary.pdf]

Supplementary materials to

Synergistic orchestration of chlorogenic acid analogues in sugarcane molasses polyphenols for sucrase inhibition: Insights into a multilevel inhibitory mechanism

Shenghong Yao [1], Wanlu Liu [1], Yumei Wang [1], Jiulong An [1], Chengfeng Zhang [2], Matthew Flavel [3, 4], Yanv Zhou [2], Lu Li [1] and He Li [1, *]

[1] Key Laboratory of Geriatric Nutrition and Health (Beijing Technology and Business University), Ministry of Education, Beijing, 100048, China

[2] The Product Makers Co., Ltd., Shanghai, 200444, China

[3] School of Life Sciences, La Trobe University, Melbourne 3083, Australia

[4] The Product Makers (Australia), Keysborough 3173, Australia

* Correspondence: lihe@btbu.edu.cn (H.L.)

1. Supplementary Tables

Table S1. UHPLC-MS/MS-based putative annotation of selected polyphenolic compounds in SMPE.

No.	Polyphenols	Relative peak area (%)	Formula	RT (min)	Precursor ion (m/z)	Major fragment ions	Annotation source(s)	mzCloud Best Match (%)	mzVault Best Match (%)
1	Neochlorogenic acid	36.36	$C_{16}H_{18}O_9$	4.428	353.0880	191.0557, 179.0345, 135.0444	mzCloud; mzVault; ChemSpider	95.8	81.5
2	Chlorogenic acid	25.70	$C_{16}H_{18}O_9$	5.248	353.0878	191.0556, 179.0345, 135.0444	mzCloud; mzVault; ChemSpider	99.4	81.4
3	Methyl chlorogenate	18.04	$C_{17}H_{20}O_9$	5.433	367.1034	193.0502, 191.0558, 134.0366	ChemSpider; Predicted Compositions	N/A	N/A
4	Vicenin III	4.19	$C_{26}H_{28}O_{14}$	6.134	563.1408	443.0986, 473.1096, 353.0668	mzVault; ChemSpider	N/A	80.6
5	Gentisic acid	3.26	$C_7H_6O_4$	3.839	153.0187	109.0286, 135.0556, 81.0343	mzCloud; mzVault; ChemSpider	94.7	82.4
6	Jaceosidin	2.26	$C_{17}H_{14}O_7$	9.859	329.0667	314.0433, 299.0197, 271.0249	mzVault; ChemSpider	N/A	72.9

Table S1. UHPLC-MS/MS-based putative annotation of selected polyphenolic compounds in SMPE.

No.	Polyphenols	Relative peak area (%)	Formula	RT (min)	Precursor ion (m/z)	Major fragment ions	Annotation source(s)	mzCloud Best Match (%)	mzVault Best Match (%)
7	Luteolin	1.34	$C_{15}H_{10}O_6$	8.991	285.0406	133.0295, 151.0033, 175.0395	mzCloud; mzVault; ChemSpider	98.3	67.4
8	Isovitexin	1.04	$C_{21}H_{20}O_{10}$	6.588	431.0983	311.0563, 341.0667, 283.0613	mzVault; ChemSpider	N/A	77.7
9	Orientin	0.89	$C_{21}H_{20}O_{11}$	6.285	447.0934	327.0511, 357.0617, 299.0402	mzVault; ChemSpider	N/A	77.1
10	Esculetin	0.86	$C_9H_6O_4$	5.712	177.0187	133.0288, 105.0337, 89.0386	mzVault; ChemSpider	N/A	76.9
11	Hispidulin	0.78	$C_{16}H_{12}O_6$	9.913	299.0562	284.0327	mzCloud; mzVault; ChemSpider	99.1	69.8
12	Isorhamnetin	0.74	$C_{16}H_{12}O_7$	8.782	315.0512	271.0614, 256.0375, 179.0344	mzCloud; ChemSpider	96.7	N/A
13	4'-O-Glucosylvitexin	0.63	$C_{27}H_{30}O_{15}$	6.036	593.1516	431.0986, 311.0563, 341.0667	mzVault; ChemSpider	N/A	55.6

Table S1. UHPLC-MS/MS-based putative annotation of selected polyphenolic compounds in SMPE.

No.	Polyphenols	Relative peak area (%)	Formula	RT (min)	Precursor ion (m/z)	Major fragment ions	Annotation source(s)	mzCloud Best Match (%)	mzVault Best Match (%)
14	Caffeic acid	0.61	$C_9H_8O_4$	4.593	179.0344	136.0159, 135.0444, 164.0473	mzCloud; mzVault; ChemSpider	66.8	52.5
15	Vicenin II	0.58	$C_{27}H_{30}O_{15}$	5.700	593.1518	503.1201, 473.1091, 383.0775	mzVault; ChemSpider	N/A	74.7
16	Phloretin	0.36	$C_{15}H_{14}O_5$	7.111	273.0769	229.0505, 214.0635, 193.0502	mzCloud; ChemSpider	96.1	N/A
17	1-Caffeoylquinic acid	0.36	$C_{16}H_{18}O_9$	2.599	353.0881	163.0395, 191.0346, 135.0445	mzVault	N/A	52.5
18	Astilbin	0.34	$C_{21}H_{22}O_{11}$	6.143	449.1091	287.0563, 151.0031, 135.0445	mzVault; ChemSpider	N/A	50.4
19	Genistein	0.32	$C_{15}H_{10}O_5$	9.776	269.0455	151.0030, 133.027, 183.0447	mzCloud; mzVault; ChemSpider	99.0	63.7

Table S1. UHPLC-MS/MS-based putative annotation of selected polyphenolic compounds in SMPE.

No.	Polyphenols	Relative peak area (%)	Formula	RT (min)	Precursor ion (m/z)	Major fragment ions	Annotation source(s)	mzCloud Best Match (%)	mzVault Best Match (%)
20	Isorhapontigenin	0.29	$C_{15}H_{14}O_4$	9.039	257.0819	242.0583, 227.0348, 213.0917	mzCloud; ChemSpider	60.6	N/A
21	Homoorientin	0.29	$C_{21}H_{20}O_{11}$	6.116	447.0934	327.0511, 357.0617, 297.0405	mzVault; ChemSpider	N/A	75.3
22	4,5-Dicaffeoylquinic acid	0.19	$C_{25}H_{24}O_{12}$	7.313	515.1196	353.0880, 173.0450, 179.0345	mzCloud; mzVault	94.2	74.4
23	Naringenin	0.19	$C_{15}H_{12}O_5$	9.813	271.0612	151.0030, 119.0494, 107.0130	mzCloud; mzVault; ChemSpider	83.0	81.8
24	Hesperidin	0.16	$C_{28}H_{34}O_{15}$	7.182	609.1833	301.0719, 151.0031, 223.0434	mzCloud; mzVault; ChemSpider	85.9	61.0
25	Naringin	0.11	$C_{27}H_{32}O_{14}$	7.159	579.1722	271.0613, 151.0030, 459.1148	mzCloud; mzVault; ChemSpider	94.4	76.2

Table S1. UHPLC-MS/MS-based putative annotation of selected polyphenolic compounds in SMPE.

No.	Polyphenols	Relative peak area (%)	Formula	RT (min)	Precursor ion (m/z)	Major fragment ions	Annotation source(s)	mzCloud Best Match (%)	mzVault Best Match (%)
26	Usnic acid	0.07	$C_{18}H_{16}O_7$	11.062	343.2125	328.0590, 313.0356, 285.0405	mzVault; ChemSpider	N/A	52.0
27	5,7,3'-Trihydroxy-6,4',5'-trimethoxyflavone	0.05	$C_{18}H_{16}O_8$	7.519	359.1137	341.1031, 329.1031, 311.0562	mzVault; ChemSpider	N/A	60.9

Note: mzCloud and mzVault Best Match values represent the spectral match values (0–100%) between the experimental MS/MS fragmentation scan and the corresponding reference spectrum in the spectral library. N/A indicates that no corresponding spectral-library match was available. mzCloud and mzVault were used as MS/MS spectral libraries, whereas ChemSpider and Predicted Compositions provided supplementary annotation evidence.

Table S2. Binding sites and interaction types of the three polyphenols with sucrase

Polyphenols	Binding energy (kcal/mol)	Hydrophobic interactions	Hydrogen bonds	π-π Stacking	Salt bridge
Chlorogenic acid	-8.0	Tyr90、Trp156、Trp163、Phe273	Tyr90、Asn91、Trp156、Phe273、Ser281	Tyr90、Trp156	-
Neochlorogenic acid	-8.2	Tyr90、Phe155、Trp156、Trp163、Phe273	Asn91、Trp156、Phe273	Tyr90、Trp156	Lys153
Methyl chlorogenate	-7.8	Trp48、Phe82、Tyr200、Trp291	Asp22、Gln40、Ser83、Gln201、Asn228	-	-

Red font indicates that two or three interactions are formed.

Table S3. Binding sites and interaction forces when multiple polyphenols are simultaneously docked with sucrase

Pair	Binding energy (kcal/mol)	Polyphenols	Hydrophobic interactions	Hydrogen bonds	π-π Stacking	Salt bridge
CA-MC	-11.9	CA	Trp48、Trp291	Asp22、Ser83、Glu203、Asn288、Ser412	Phe82	Lys385
		MC	Tyr90、Trp156、Trp163、Ile208、Phe273	Phe273、Ser281	Tyr90、Trp156	-
CA-NA	-11.5	CA	Glu209、Asn274	Tyr90、Glu209、Ser219、Ser281	-	-
		NA	Tyr90、Phe155、Trp156、Trp163、Phe273	Asn91、Glu158、Glu209	Tyr90、Trp156	Lys153
NA-MC	-11.5	NA	Trp291、Phe82	Asn114、Thr147、Gln148、Arg150、Gln201、Ser412、Ala413	-	Arg150
		MC	Trp48、Phe82、Trp291	Asn21、Gln40、Arg150、Lys385、Ser412	-	Lys385
CA-NA-MC	-14.0	CA	Tyr90、Trp156、Trp163、Phe273	Asn91、Trp156、Phe273、Ser281	Tyr90、Trp156	-
		NA	Tyr90、Asn274	Glu30、Tyr90、Asn91、Asn274、Thr275	Tyr90	-
		MC	Phe82、Trp291	Ser83、Arg150、Ser412	-	Lys385

Red font indicates that two or three interactions are formed.

Table S4. Kinetic inhibition parameters of sucrase in the presence of CA, NA, and MC

Inhibitor	[I] (mg/mL)	V_{max} (ΔOD/min)	K_m (mg/mL)	R^2	K_i (mg/mL)	α	Sy.x
CA	0	0.248 ± 0.028[a]	14.326 ± 2.055[b]	0.9872	9.788 ± 1.021	-	0.06676
	3.0	0.245 ± 0.008[a]	16.865 ± 0.191[b]	0.9778			
	3.5	0.273 ± 0.056[a]	23.461 ± 6.925[ab]	0.9851			
	4.0	0.266 ± 0.043[a]	26.009 ± 6.401[a]	0.9815			
NA	0	0.257 ± 0.056[a]	17.858 ± 4.967[c]	0.9643	3.489 ± 0.369	-	0.06066
	2.5	0.259 ± 0.041[a]	26.104 ± 7.098[b]	0.9612			
	3.0	0.311 ± 0.010[a]	42.065 ± 1.249[a]	0.9992			
	3.5	0.257 ± 0.049[a]	45.326 ± 3.767[a]	0.9866			
MC	0	0.173 ± 0.006[a]	7.316 ± 0.977[b]	0.9745	12.160 ± 2.008	2.43	0.02707
	4.00	0.156 ± 0.005[b]	9.618 ± 0.310[a]	0.9798			
	4.75	0.153 ± 0.007[b]	10.222 ± 0.695[a]	0.9962			
	5.50	0.152 ± 0.020[b]	10.591 ± 2.786[a]	0.9792			

"-" indicates that the parameter is not applicable, because CA and NA act as competitive inhibitors, and therefore the value of α cannot be determined. Different lowercase letters indicate significant differences among concentrations ($p < 0.05$).

Table S5. Kinetic inhibition parameters of sucrase in the presence of SMPE

[I] (mg/mL)	V_{max} (ΔOD/min)	K_m (mg/mL)	R^2	K_i (mg/mL)	α
0	0.106 ± 0.010[a]	14.873 ± 2.353[a]	0.9638	-	-
3	0.077 ± 0.009[b]	12.382 ± 1.693[ab]	0.9107		
4	0.049 ± 0.011[c]	9.864 ± 3.410[b]	0.9359		
5	0.037 ± 0.005[c]	7.821 ± 2.634[b]	0.9325		

"-" indicates that the parameter is not applicable. Because SMPE contains multiple active constituents and exhibits complex inhibitory behavior, the meanings of K_i and α are not clearly defined and therefore were not calculated. Different lowercase letters indicate significant differences among concentrations ($p < 0.05$).

R^2 represents the coefficient of determination for the linear regression of the Lineweaver-Burk plots, whereas Sy.x represents the standard deviation of the residuals for the nonlinear regression used to estimate K_i and α.

Table S6. RMSF values of selected amino acid residues in sucrase before and after binding with polyphenols

Polyphenols	Residue	Sucrase (nm)	Sucrase-Polyphenol (nm)
CA	Tyr90	0.1815	0.0775
	Asn91	0.1058	0.1062
	Trp156	0.0828	0.0794
	Trp163	0.0656	0.0647
	Phe273	0.0665	0.0585
	Ser281	0.1289	0.1378
NA	Tyr90	0.1815	0.2060
	Asn91	0.1058	0.1098
	Lys153	0.0682	0.0687
	Phe155	0.0693	0.0651
	Trp156	0.0828	0.0761
	Trp163	0.0656	0.0613
	Phe273	0.0665	0.0728
MC	Asp22	0.0777	0.0761
	Gln40	0.1010	0.0996
	Trp48	0.1294	0.1504
	Phe82	0.1290	0.1008
	Ser83	0.0918	0.0704
	Tyr200	0.0710	0.0710
	Gln201	0.0839	0.0773
	Asn228	0.0802	0.1018
	Trp291	0.0696	0.0757

2. Supplementary Figures

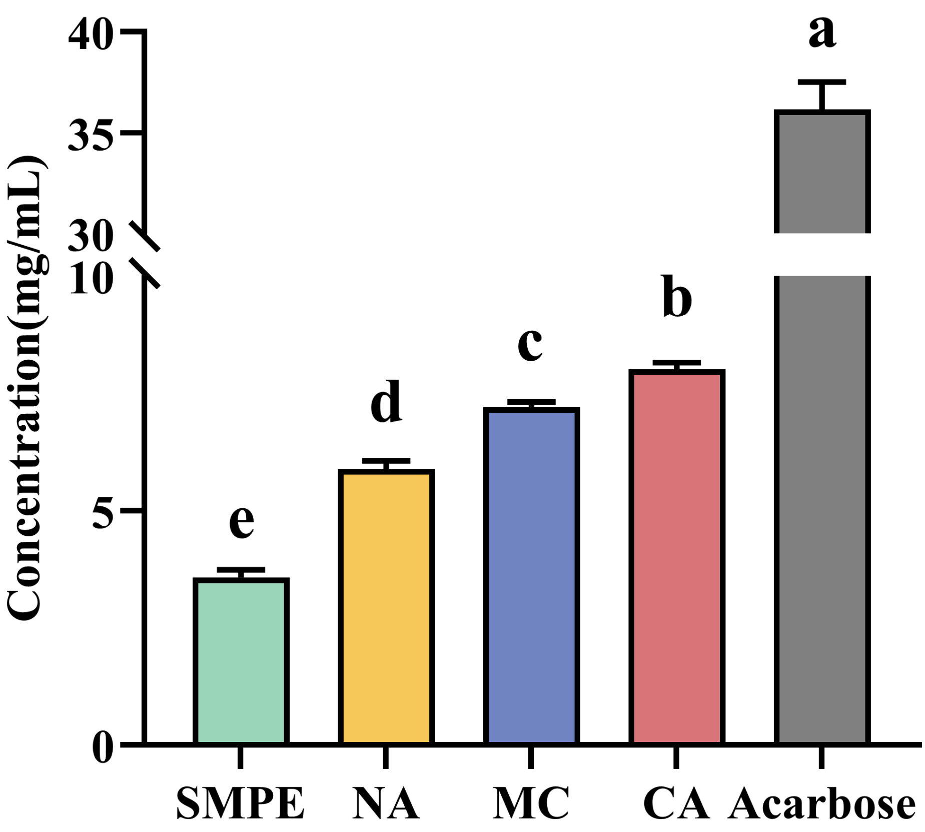

Figure S1. Comparison of IC_{50} values of sucrase for four polyphenols and acarbose. Bars labeled with different lowercase letters indicate significant differences at $p < 0.05$.

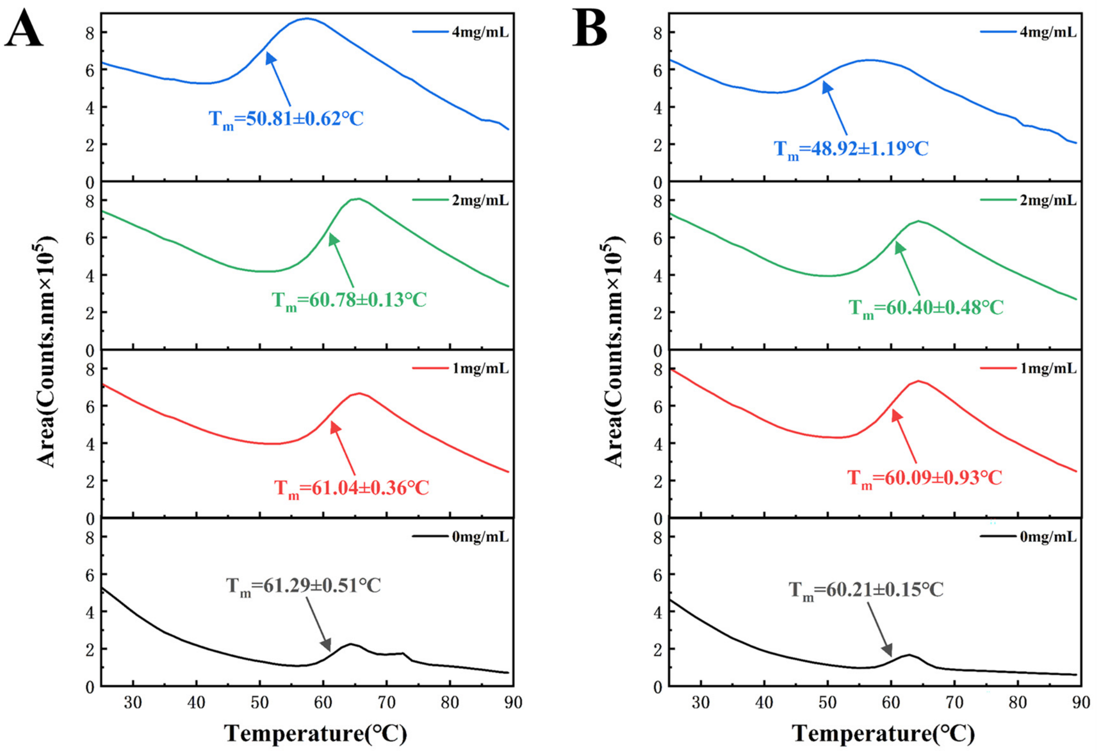

Figure S2. Fluorescence peak area of sucrase as a function of temperature after interaction with different concentrations of CA (A) and NA (B).
